# Four magnetic resonance imaging surveillance-detected breast cancer cases in cancer-free *BRCA1/2* mutation carriers

**DOI:** 10.1186/s40792-021-01313-5

**Published:** 2021-10-21

**Authors:** Megumi Takaoka, Shozo Ohsumi, Yuichiro Miyoshi, Mina Takahashi, Seiki Takashima, Kenjiro Aogi, Teruhiko Shimizu, Norihiro Teramoto, Yasuko Yamamoto, Miki Okamura

**Affiliations:** 1 Department of Breast Oncology, National Hospital Organization Shikoku Cancer Center, 160 Kou, Minami-umenomoto-machi, Matsuyama, 791-0280 Japan; 2 Department of Radiology, National Hospital Organization Shikoku Cancer Center, 160 Kou, Minami-umenomoto-machi, Matsuyama, 791-0280 Japan; 3 Department of Pathology, National Hospital Organization Shikoku Cancer Center, 160 Kou, Minami-umenomoto-machi, Matsuyama, 791-0280 Japan; 4 Department of Familial Tumor, National Hospital Organization Shikoku Cancer Center, 160 Kou, Minami-umenomoto-machi, Matsuyama, 791-0280 Japan

**Keywords:** *BRCA*, Hereditary breast and ovarian cancer syndrome, MRI surveillance, Breast cancer

## Abstract

**Background:**

Hereditary breast and ovarian cancer (HBOC) syndrome is a susceptibility syndrome for cancers, such as breast and ovarian cancer, and *BRCA1/2* are its causative genes. Annual breast-enhanced magnetic resonance imaging (MRI) is recommended for *BRCA1/2* mutation carriers aged over 25 years as a secondary prevention of breast cancer. However, breast MRI surveillance is rarely performed in Japan, and only four cases of breast cancer diagnosis triggered by MRI surveillance have been reported.

**Case presentation:**

At our hospital, MRI triggered the diagnosis of breast cancer in four cancer-free *BRCA1/2* mutation carriers. In one of our four cases, although MRI showed only a 3-mm focus, we could diagnose breast cancer by shortening the surveillance interval considering the patient’s high-risk for developing breast cancer.

**Conclusions:**

Image-guided biopsy, including MRI-guided biopsy, depending on the size of the lesion, and shorter surveillance intervals are useful when there are potentially malignant findings on breast MRI surveillance for cancer-free patients with HBOC.

## Background

Among all breast cancers, 5–10% are hereditary, and *BRCA1/2* are widely known as representative genes that can cause hereditary breast and ovarian cancer (HBOC) syndrome. The cumulative breast cancer risk for mutation carriers at the age of 70 years is 57% for *BRCA1* and 49% for *BRCA2* mutation carriers, with a high rate of developing breast cancer [1]. The mean age at diagnosis of *BRCA1/2*-associated breast cancer is much lower and this cancer has biologically aggressive phenotypes [2]. The National Comprehensive Cancer Network (NCCN) Guidelines recommend annual breast screening magnetic resonance imaging (MRI) with contrast for women aged 25–29 years, and annual mammography (MMG) that should be added for those aged 30–75 years [3]. We conducted surveillance for women with a *BRCA* pathogenic variant in accordance with the NCCN guidelines.

In Japan, breast MRI surveillance for women with a *BRCA* pathogenic variant who have not developed breast and ovarian cancer is not covered by the National Medical Insurance; therefore, surveillance is rarely performed. Only few cases of breast cancer diagnosed by breast MRI in cancer-free women with HBOC have been reported in Japan. We performed breast MRI surveillance in women with HBOC who were free of breast and ovarian cancer, and detected four breast cancers. We report these patients along with a review of the literature.

## Case presentation

### Case 1

**Fig. 1:**
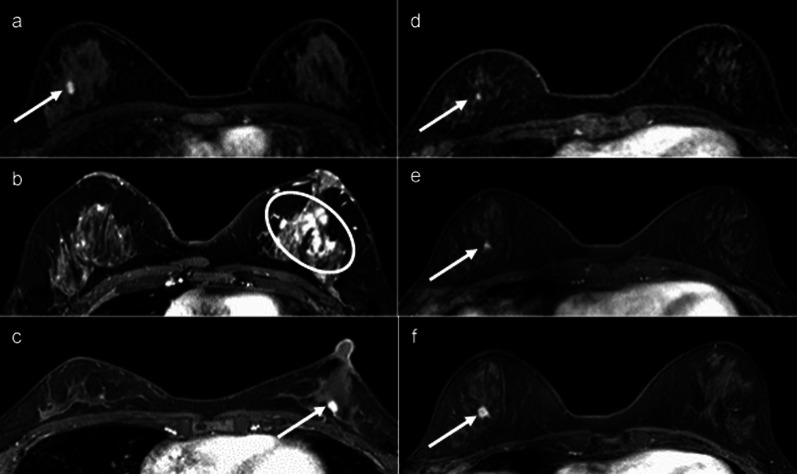
Fat-suppressed post contrast T1-weighted image. **a** A 9-mm oval circumscribed mass (arrow of **a**) on Case 1. **b** Segmental clumped non-mass enhancement (circle of **b**) on Case 2. **c** A 6-mm round circumscribed mass on the chest wall side (arrow of **c**) on Case 3. **d** A 3-mm enhanced nodule (focus) (arrow of **d**) on Case 4. **e** Three months later from first screening MRI, no significant change (arrow of **e**) on Case 4. **f** Nine months later from first screening MRI, the lesion was increased as a 10-mm not-circumscribed and heterogeneous enhanced mass (arrow of **f**) on Case 4

**Fig. 2:**
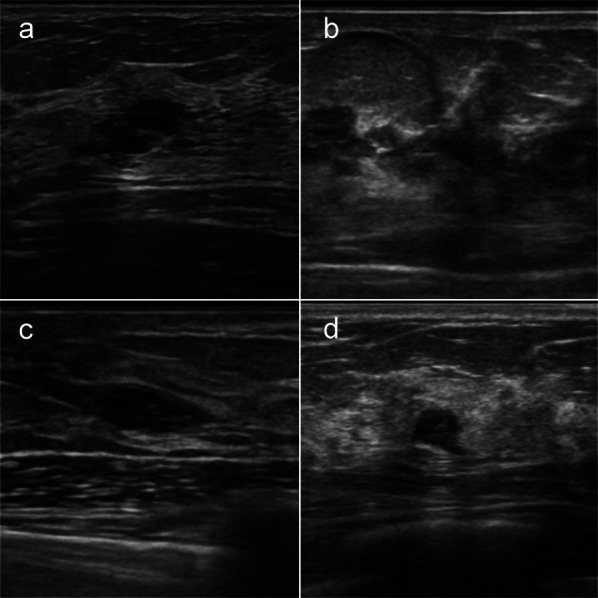
Ultrasonographic image. **a** Dilated duct collection on Case 1. **b** Distortion on Case 2. **c** A circumscribed lobulated hypoechoic mass on Case 3. **d** A microlobulated irregular hypoechoic mass on Case 4

**Fig. 3:**
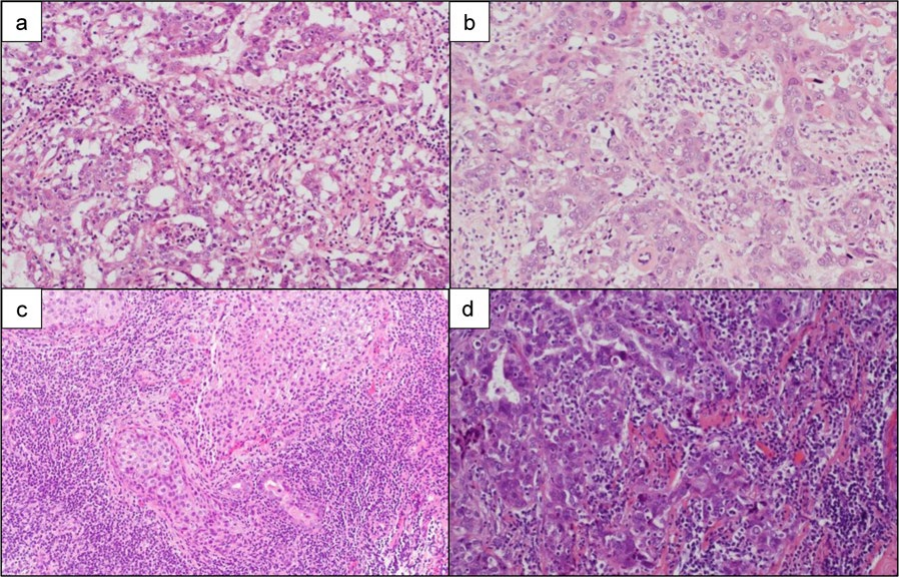
Microscopic hematoxylin and eosin photograph. **a** Invasive ductal carcinoma that grows like a follicle on Case 1. **b** Invasive ductal carcinoma scirrhous type on Case 2. **c** Microinvasive ductal carcinoma on Case 3. **d** Invasive ductal carcinoma solid type on Case 4

A 26-year-old woman with a *BRCA1* pathogenic variant underwent annual breast screening MRI four times over 3 years. The fourth screening MRI showed a 9-mm oval circumscribed mass (Fig. 1a). MRI-targeted ultrasonography (US) showed dilated duct collection (Fig. 2a), and we performed US-guided core needle biopsy (CNB). With a triple-negative invasive ductal carcinoma diagnosis, the patient underwent total mastectomy, sentinel lymph node biopsy, and tissue expander reconstruction. The tumor was pT1bN0M0 stage I (Fig. 3a).

### Case 2

A 36-year-old woman with a *BRCA2* pathogenic variant underwent annual breast screening MRI eight times over 7 years. The first to eighth screening MRI showed a lot of punctate and nodular enhancement in the bilateral breasts. The eighth screening MRI showed segmental clumped non-mass enhancement on the lateral side of the left breast for the first time (Fig. 1b). MRI-targeted US showed distortion (Fig. 2b) and axillary lymphadenopathy. US-guided CNB and lymph node fine-needle aspiration were performed on the patient. With a diagnosis of triple-negative invasive ductal carcinoma and axillary lymph node metastasis, she underwent total mastectomy and level I axillary lymph node dissection. The tumor was pT1bN1M0 stage IIA (Fig. 3b). She received chemotherapy in a clinical trial. Two years after breast cancer surgery, the patient underwent risk-reducing salpingo-oophorectomy (RRSO).

### Case 3

A 43-year-old woman with a *BRCA2* pathogenic variant underwent breast screening MRI for the first time. The first screening MRI showed a 6-mm round circumscribed enhanced mass on the chest wall side (Fig. 1c). The enhancement pattern was fast-washout, and diffusion-weighted imaging (DWI) (*b* = 1500) showed a high signal. MRI-targeted US showed a circumscribed lobulated hypoechoic mass (Fig. 2c), and we performed US-guided CNB on the patient. She underwent total mastectomy and sentinel lymph node biopsy for a diagnosis of luminal invasive ductal carcinoma. The tumor was pT1miN0M0 stage I (Fig. 3c). At the same time as breast cancer surgery, the patient underwent RRSO.

### Case 4

A 49-year-old woman with a *BRCA2* pathogenic variant underwent breast screening MRI for the first time. The first screening MRI showed a 3-mm enhanced nodule (focus) (Fig. 1d). Since she was at high risk of developing breast cancer and the lesion was presumed to be malignant, breast MRI was performed again 3 months later. No significant changes were observed (Fig. 1e) in the patient. Nine months after the first screening MRI, breast MRI was performed once again, and the lesion had increased to 10 mm. It was a non-circumscribed and heterogeneously enhanced mass (Fig. 1f). The enhancement pattern was fast-washout, and DWI (*b* = 1500) showed a high signal. MRI-targeted US showed a microlobulated irregular hypoechoic mass (Fig. 2d), and we performed US-guided CNB on the patient. She underwent partial mastectomy and sentinel lymph node biopsy for a diagnosis of luminal invasive ductal carcinoma. Subsequently, she underwent radiotherapy of the breast. The tumor was pT1bN0M0 stage I (Fig. 3d).

## Discussion

According to the EVA trial, which was a prospective multicenter observational cohort study conducted to compare breast cancer detection rates for MMG, US, and MRI in high-risk individuals with breast cancer, the cancer yields were 5.4/1000, 6.0/1000, and 14.9/1000 women, respectively. The cancer yield achieved by MRI was significantly higher [4]. In a German study that aimed to evaluate the efficacy of US for the early detection of breast cancer in *BRCA1/2* carriers screened by semi-annual US in combination with annual MMG and MRI, 3 of 27 (11.1%) detected tumors were found in the semi-annual US. Semi-annual US performed during annual MRI was thus useful [5]. At our hospital, we recommend an annual breast screening MRI with contrast for patients with HBOC. For those who gave their consent, we conducted annual MRI, and MMG and US between MRI surveillance.

In Japan, *BRCA1/2* genetic testing has been covered by the National Medical Insurance only for companion diagnostics for patients with HER2-negative recurrent breast cancer since 2018. From April 2020, it has also been covered by insurance for HBOC diagnostics for patients who meet the conditions, such as onset under 45 years of age or those having a family history of breast cancer. At the same time, risk-reducing mastectomy and contrast-enhanced breast MRI surveillance for *BRCA1/2* mutation carriers developing breast or ovarian cancer are also covered by insurance. However, contrast-enhanced breast MRI surveillance for *BRCA1/2* mutation carriers who have not developed breast and ovarian cancer is not covered by insurance; therefore, breast MRI surveillance is rarely performed. We searched on PubMed using the keywords “*BRCA*” and “MRI” to examine the cases that led to the diagnosis of breast cancer by breast MRI surveillance of cancer-free patients with HBOC in Japan. Four cases were found accordingly (Table 1) [6–8]. When combined with our four cases, six of the eight cases could be identified by MRI-targeted US. Most of them showed only category 2 findings, which normally require no examination. In particular, *BRCA1*-associated breast cancer often appears as a fibroadenoma or cyst on US [9]. For high-risk patients, it is necessary to actively perform a biopsy to make a diagnosis when there are some findings on MRI, even if the US shows benign findings. In Case 4, as the first MRI showed only a 3-mm focus that could not be identified by US and it was too small to perform an MRI-guided biopsy, we decided to follow-up with MRI 3 months later. No significant changes were observed, and we performed MRI once again 9 months after the first screening MRI. The lesion had increased, and we could diagnose breast cancer by the third screening MRI.

**Table 1:** Reported cases of breast cancer detected by MRI on cancer-free HBOC patients in Japan

No	Year	Author	Age at breast cancer diagnosis	Mutation	Surveillance period (month)	Times of MRI surveillance	MRI findings	MRI-targeted US findings	Stage
1	2021	Our Case 1	27	*BRCA1*	39	4	Mass	Dilated duct collection	pT1bN0M0 StageI
2	2021	Our Case 2	43	*BRCA2*	86	8	Non-mass	Distortion	pT1bN1M0 StageIIA
3	2021	Our Case 3	43	*BRCA2*	0	1	Mass	Mass	pT1miN0M0 StageI
4	2021	Our Case 4	53	*BRCA2*	42	3	Mass	Mass	pT1bN0M0 StageI
5	2019	Shimada [6]	35	*BRCA1*	145	1	Non-mass	N/A	pTisN0M0 Stage0
6	2019	Shimada [6]	48	*BRCA2*	51	1	Non-mass	N/A	pTisN0M0 Stage0
7	2019	Murakami [7]	N/A	*BRCA1*	N/A	1	Mass	Visible lesion	N/A
8	2017	Tozaki [8]	48	*BRCA2*	N/A	N/A	Non-mass	hypoechoic area	pTisN0M0 Stage0

*MRI* magnetic resonance imaging, *US* ultrasonography, *N/A* not applicable

Breast MRI with contrast has been reported to show a round or oval mass on the chest wall side in *BRCA1*-associated breast cancer [10] and non-mass enhancement in *BRCA2*-associated breast cancer [7]. In addition, MMG has been reported to show calcification only in *BRCA2*-associated breast cancer [7]. In our cases, these features were relevant to Cases 1 and 2, but not for Cases 3 and 4. Understanding the features of these images may increase the detection rate of breast cancer and may be useful in examining surveillance intervals accordingly.

Recently, it has been reported that among patients with *BRCA1/2* pathogenic or likely pathogenic variants, high-risk clinicopathological factors, and HER2-negative breast cancer, adjuvant olaparib improved the 3-year invasive disease-free survival (85.9% in the olaparib group and 77.1% in the placebo group; hazard ratio for invasive disease or death, 0.58; 99.5% CI 0.41 to 0.82; *P* < 0.001) [11]. Given this result, the importance of diagnosing breast cancer in patients with HBOC must increase. In two of the four previously reported cases, the first breast MRI was performed 51 months and 145 months after starting surveillance, and both cases resulted in a diagnosis of breast cancer triggered by the first breast MRI. At present, routine breast MRI surveillance for cancer-free patients with HBOC is still challenging in Japan, but it is still considered useful.

## Conclusions

Image-guided biopsy, including MRI-guided biopsy depending on the size of the lesion, and shorter surveillance intervals, are useful when there are potentially malignant findings on breast screening MRI with contrast for cancer-free women with HBOC.

## Data Availability

Not applicable.

## Abbreviations

HBOC: Hereditary breast and ovarian cancer
MRI: Magnetic resonance imaging
NCCN: National Comprehensive Cancer Network
MMG: Mammography
US: Ultrasonography
CNB: Core needle biopsy
RRSO: Risk-reducing salpingo-oophorectomy
DWI: Diffusion-weighted imaging
